# Dipolar skyrmions and antiskyrmions of arbitrary topological charge at room temperature

**DOI:** 10.1038/s41567-023-02358-z

**Published:** 2024-01-25

**Authors:** Mariam Hassan, Sabri Koraltan, Aladin Ullrich, Florian Bruckner, Rostyslav O. Serha, Khrystyna V. Levchenko, Gaspare Varvaro, Nikolai S. Kiselev, Michael Heigl, Claas Abert, Dieter Suess, Manfred Albrecht

**Affiliations:** 1 Institute of Physics, University of Augsburg, Augsburg, Germany; 2 ISM – CNR, nM2-Lab, Monterotondo Scalo, Roma, Italy; 3 Physics of Functional Materials, Faculty of Physics, University of Vienna, Vienna, Austria; 4 Vienna Doctoral School in Physics, University of Vienna, Vienna, Austria; 5 Research Platform MMM Mathematics – Magnetism – Materials, University of Vienna, Vienna, Austria; 6 Nanomagnetism and Magnonics, Faculty of Physics, University of Vienna, Vienna, Austria; 7 Peter Grünberg Institute and Institute for Advanced Simulation, Forschungszentrum Jülich and JARA, Jülich, Germany

**Keywords:** Nanoscale materials, Nanoscale materials, Physics, Magnetic properties and materials

## Abstract

Magnetic skyrmions are localized, stable topological magnetic textures that can move and interact with each other like ordinary particles when an external stimulus is applied. The efficient control of the motion of spin textures using spin-polarized currents opened an opportunity for skyrmionic devices such as racetrack memory and neuromorphic or reservoir computing. The coexistence of skyrmions with high topological charge in the same system promises further possibilities for efficient technological applications. In this work, we directly observe dipolar skyrmions and antiskyrmions with arbitrary topological charge in Co/Ni multilayers at room temperature. We explore the dipolar-stabilized spin objects with topological charges of up to 10 and characterize their nucleation process, their energy dependence on the topological charge and the effect of the material parameters on their stability. Furthermore, our micromagnetic simulations demonstrate spin-transfer-induced motion of these spin objects, which is important for their potential device application.

## Abstract (web summary)

Control over magnetic skyrmions at room temperature has important applications in technology. Now the observation of skyrmions with high topological charge widens the potential for them to be used in unconventional computing techniques.

## Main

Over the past few years, topological magnetic textures with particle-like properties have attracted great attention. The magnetization of these structures can be quantified by the invariant known as the topological charge *Q*. The magnetic textures for which *Q* is an integer number are called topologically protected. Magnetic skyrmions (SKs) are a prominent example of such stable topological excitations^1–4^. The topological protection of the SKs means that their spin texture cannot be continuously transformed into a saturated ferromagnetic state, that is without the appearance of singularities in the magnetization field.

There are two fundamentally different mechanisms for SK stabilization. The first one is based on short-range interactions, for example, magnetic SKs in chiral magnets emerging from the competition between the Heisenberg exchange and the Dzyaloshinskii–Moriya interaction^3–10^. The second introduces a handedness in the system, thus the chirality. Most chiral magnets host only SKs with *Q* = −1. There are only a few systems in which SK bundles and SK bags of arbitrary charge have been reported both theoretically^11–13^ and experimentally^14–18^^,^. The presence of these so-called high-*Q* SKs promises new and rich physics. However, their host system comes with certain disadvantages. One is that, in these materials, cryogenic temperatures are usually required for SK stability. Moreover, chiral magnets with bulk Dzyaloshinskii–Moriya interaction need to be grown as single crystals, which may prevent industrial scaling for fast adaptations.

The second main mechanism is based on the competition between long-range dipolar interaction and short-range exchange interactions. In this case, thin ferrimagnetic films exhibiting a perpendicular magnetic anisotropy can host dipolar-stabilized SKs, as recently reported^19,20^, whereby the interplay of low saturation magnetization and low perpendicular magnetic anisotropy leads to the stabilization of SKs. One key advantage is that they can be fabricated by magnetron sputtering at room temperature, offering a very accessible fabrication method that has already been in industrial use for many decades. Furthermore, the thickness and material of the individual layers can be easily adjusted to achieve the desired magnetic properties. For instance, by tuning the material parameters and reducing the saturation magnetization and anisotropy constant even further, magnetic SKs (*Q* = −1), trivial type-II bubbles (*Q* = 0) and antiskyrmions (ASKs) (*Q* = 1) are already reported to be stable and coexist^21^. A very rare spin object was also observed in Fe/Gd-based multilayers: an ASK with *Q* = 2, disclosing an extra iteration of Bloch and Néel walls, referred to in refs. ^21,22^ as second-order ASK. This ASK was smaller than 200 nm. Only a few studies exist that theoretically describe extra iterations of Bloch and Néel segments in dipolar-stabilized hard magnetic bubbles^23^, which were intensively investigated in the 1970s for magnetic storage applications. However, their sizes exceeded the typical dipolar-SK sizes, forming rather large and irregular domain structures. Notably, large bubble domain walls containing Néel sections, also known as vertical Bloch lines^24^ (VBLs), resembling the *Q* = 2 ASK were reported in bubble domain materials^25,26^, such as in amorphous GdCoAu alloys.

In this work, we report on the direct observation of dipolar-stabilized SKs and ASKs of small size and arbitrary topological charge at room temperature in Co/Ni multilayers. To investigate these magnetic solitons, in which the higher topological charge originates from extra iterations of Bloch and Néel segments, multilayers with different repetition numbers were fabricated. Magnetic textures were investigated using Lorentz transmission electron microscopy (LTEM). Accompanying micromagnetic simulations were performed that showed the magnetic configuration and allowed an in-depth characterization of the magnetic solitons, and of their topological charge. Although the size of these spin objects increases with their topological charge, their average size stays below 500 nm, and can be tuned with the external magnetic fields. Furthermore, current-induced motion based on spin-transfer torque of various spin objects is demonstrated by micromagnetic simulations. The very accessible fabrication method, and their stability at room temperature, establishes a new platform for both fundamental and applied research of dipolar SKs and ASKs of arbitrary topological charge. This material system and the hosted magnetic textures, reported in this work, provide extra degrees of freedom enabling different applications ranging from unconventional computing^27^ to new storage concepts^28^, and deliver an important contribution to the emerging field of skyrmionics^29^.

## High-order dipolar-stabilized SKs and ASKs

**Fig. 1:**
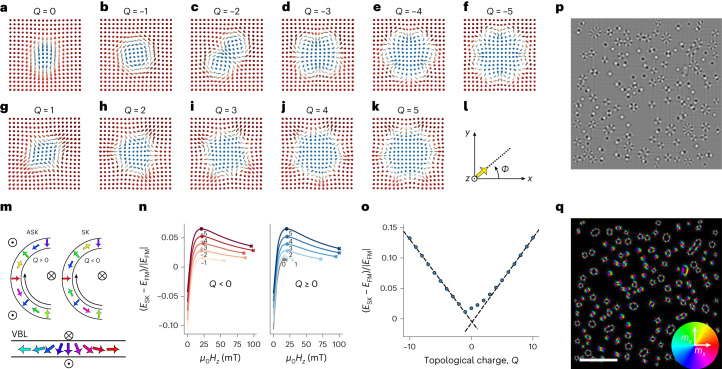
Dipolar SKs and ASKs of arbitrary topological charge. **a**–**k**, Spin configurations of dipolar-stabilized SKs with topological charges *Q* = 0 (**a**), *Q* = −1 (**b**), *Q* = −2 (**c**), *Q* = −3 (**d**), Q = −4 (**e**) and *Q* = −5 (**f**), and ASKs with *Q* = 1 (**g**), *Q* = 2 (**h**), *Q* = 3 (**i**), *Q* = 4 (**j**) and *Q* = 5 (**k**). **l**,**m**, The ASKs are defined using the coordinates system in **l**, and the counterclockwise rotation of the magnetization along the soliton boundary depicted in **m** (left), whereas the SKs are defined by a clockwise rotation of magnetization (right). The colour of arrows is given by the colour wheel in **q**. **n**,**o**, Dependence of the energy *E*_SK_ of SKs (left) and ASKs (right) of higher charge (with respect to the energy *E*_FM_ of the ferromagnetic state) on the applied OOP magnetic field (**n**), from which the dependence between the (A)SK energy and topological charge (**o**) is obtained using values at a fixed magnetic field (*μ*_0_*H*_*z*_ = 15 mT). **p**,**q**, The performed large-scale magnetic simulations are illustrated using the calculated underfocused LTEM image (**p**) and the magnetic induction map (**q**), disclosing the coexistence of SKs and ASKs with arbitrary topological charge. Scale bar, 1 µm. Source data

In this section, we aim to describe the magnetic configuration of the new spin textures. To differentiate these spin textures from the SK bags or bundles in chiral magnets, we introduce the terminology of high-order (A)SKs. Figure 1 gives a first overview of these spin objects that were observed in micromagnetic simulations (Methods) using the graphics processing unit accelerated micromagnetic code magnum.np^30^ (https://gitlab.com/magnum.np/magnum.np). As seen, these (A)SKs are enclosed by one single domain boundary, which contains an arbitrary number of Bloch and Néel segments, resulting in higher topological charges. Dipolar (A)SKs with topological charges of between *Q* = −5 and 5 are illustrated in Fig. 1a–k. For the remainder of the article, we will drop the word dipolar as it should be clear by now what type of spin objects we are dealing with. The spin configurations are obtained as stable magnetization states from our micromagnetic simulations. Using standard parametrization of the magnetization unit vector field ${\mathbf{m}}={\left(\cos \left(\varPhi \right)\sin \left(\varTheta \right),\sin \left(\varPhi \right)\sin \left(\varTheta \right),\cos \left(\varTheta \right)\right)}^{T}$, one can calculate the topological charge of the given magnetization states using the contour integral^13^

1 $$Q=\frac{1}{2\pi }\int \nabla \varPhi \cdot \rm{d}\it{r}$$

where *Φ* is the azimuthal angle of magnetization (Fig. 1l) and integration is over the oriented contour with polar angle, *Θ* = π/2. Using equation (1) together with the schematic magnetic configurations at the soliton boundary (*Θ* = π/2) given in Fig. 1m, one can then define the high-order SKs and ASKs as follows. When moving along the contour (Fig. 1m), the angle *Φ* rotates counterclockwise, that is, in the mathematical positive direction, we have ASKs with *Q* > 0 (Fig. 1m, left). If *Φ* rotates clockwise, then the resulting spin textures are SKs with *Q* < 0 (Fig. 1m, right). *Q* = 0 still corresponds to trivial type-II bubbles. As will be discussed in more detail later, the presence of VBLs illustrated in Fig. 1m in the (A)SK boundaries will have a crucial role in the formation process of the high-order (A)SKs.

The self-energy of (A)SKs as a function of the external magnetic field is presented in Fig. 1n. It is seen that, in most of the range of their stability, high-order (A)SKs have higher energies than the saturated ferromagnetic state. There exists a magnetic field for which the high-order (A)SKs have their highest energy overall. If we compare the energies at a fixed external field of 15 mT, one can observe (Fig. 1o) that, overall, the energy of the SKs and ASKs increases nearly linearly with increasing topological charge. Similar to SK bags in chiral magnets, the topological charge and the size of dipolar SKs are limited only by the physical size of the system. On the other hand, contrary to chiral magnets, the dipolar high-order SKs have energies that are nearly identical to those of their topological counterpart and the small difference even reduces with increasing *Q* (see dashed lines in Fig. 1o).

**Extended Data Fig. 1:**
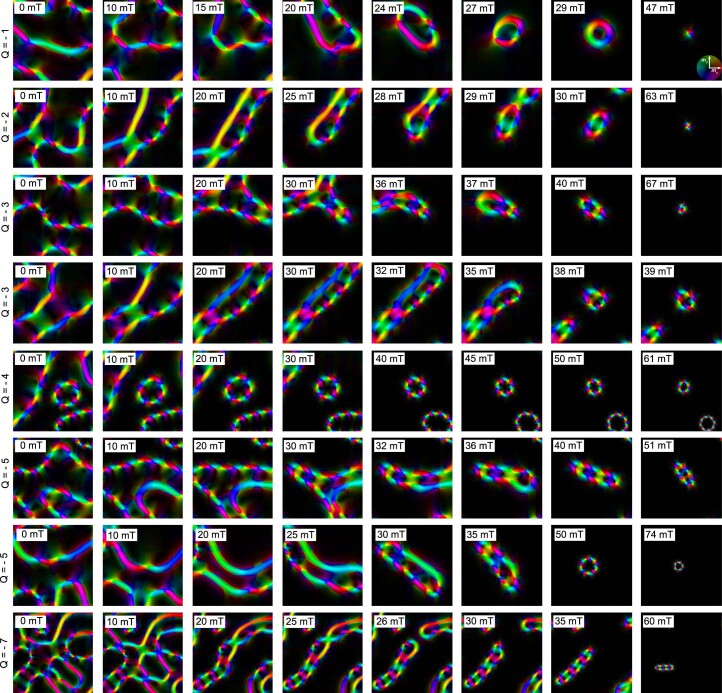
Nucleation of high-order skyrmions. Magnetic induction maps illustrating the field dependence of the nucleation process of SKs up to *Q* = −7. The used film thickness and material parameters correspond to the [Co/Ni]_10_ multilayer sample. The color-wheel illustrates the in-plane projection of magnetization.

Our numerical investigations show that a large variety of high-order (A)SKs can coexist. To illustrate that, we relax a magnetic thin film (homogeneous along the *z* axis) starting from a randomly magnetized state, and apply an out-of-plane (OOP) magnetic field (Methods). A small section of the simulated structure is presented as an LTEM contrast image (Fig. 1p) and an induction map (Fig. 1q), where the coexistence of SKs and ASKs with arbitrary topological charge can be observed. The ASKs are found to be almost always in a circular magnetization state, elongating only with increasing *Q*, whereas the SKs with *Q* < −2 appear mostly with two different symmetries: circular and elongated. Note that an elongated SK with *Q* = −2 could be called a biskyrmion and that with *Q* = −3 a triskyrmion^31^. The circular SKs are illustrated in Fig. 1a; by contrast, the elongated high-order SKs resemble a chain of interconnected single SKs (Extended Data Fig. 1). Nevertheless, they are still constrained by one single domain boundary or single contour according to ref. ^13^.

## Direct observation of high-order SKs and ASKs

**Fig. 2:**
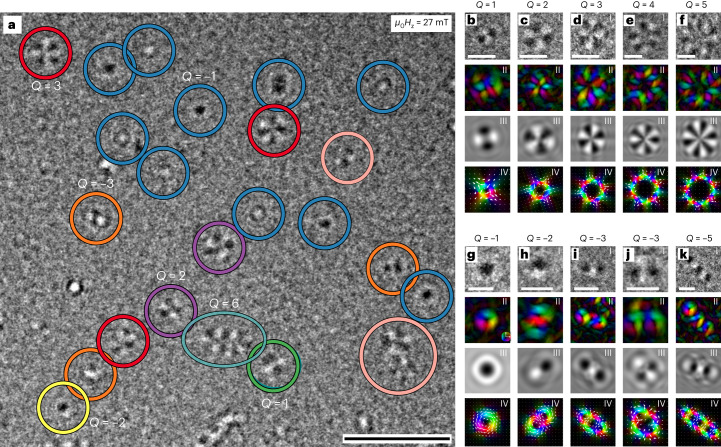
High-order SKs and ASKs at room temperature. **a**, LTEM image of a [Co/Ni]_10_ multilayer sample taken in an applied OOP magnetic field of 27 mT showing different spin objects with |*Q*| values up to 6. Some non-classified spin objects are marked with pink circles. **b**–**k**, (I) Zoomed-in LTEM images showing individual ASKs for |*Q*| = 1 (**b**), |*Q*| = 2 (**c**), |*Q*| = 3 (**d**), |*Q*| = 4 (**e**) and |*Q*| = 5 (**f**), and SKs for |*Q*| = −1 (**g**), |*Q*| = −2 (**h**), |*Q*| = −3 (**i**), |*Q*| = −4 (**j**) and |*Q*| = −5 (**k**). Note that circular and elongated SKs with *Q* = −3 can exist. (II) Corresponding induction maps. (III) Simulated Lorentz contrast. (IV) Simulated induction maps. Scale bars, 1 µm (**a**), 200 nm (**b**–**k**).

**Extended Data Fig. 2:**
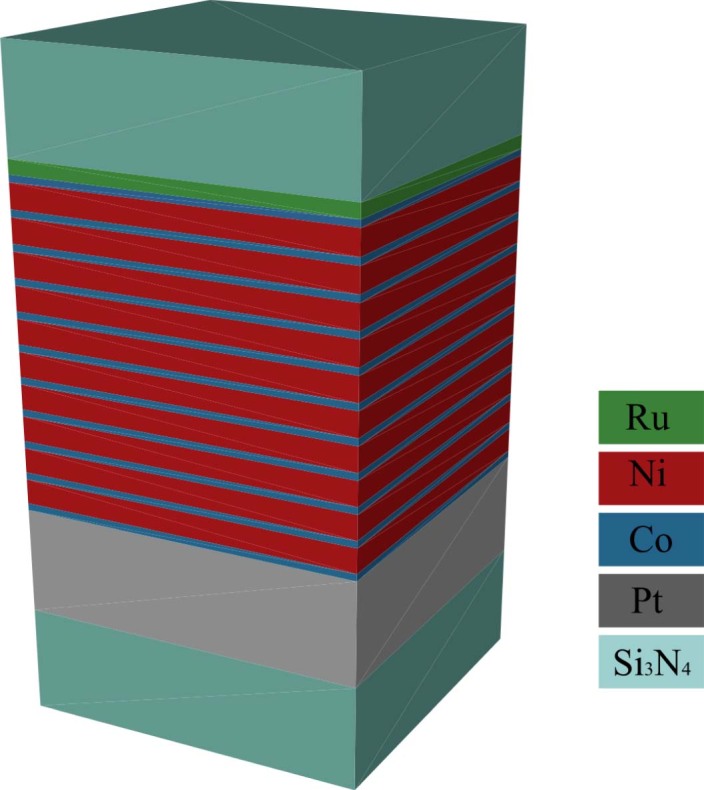
Schematic illustration of the multilayer stack. Three dimensional representation of the [Co/Ni]_N_ multilayers consisting of Co(0.2 nm)/Ni(0.7 nm) bilayers being repeated N times.

**Extended Data Fig. 3:**
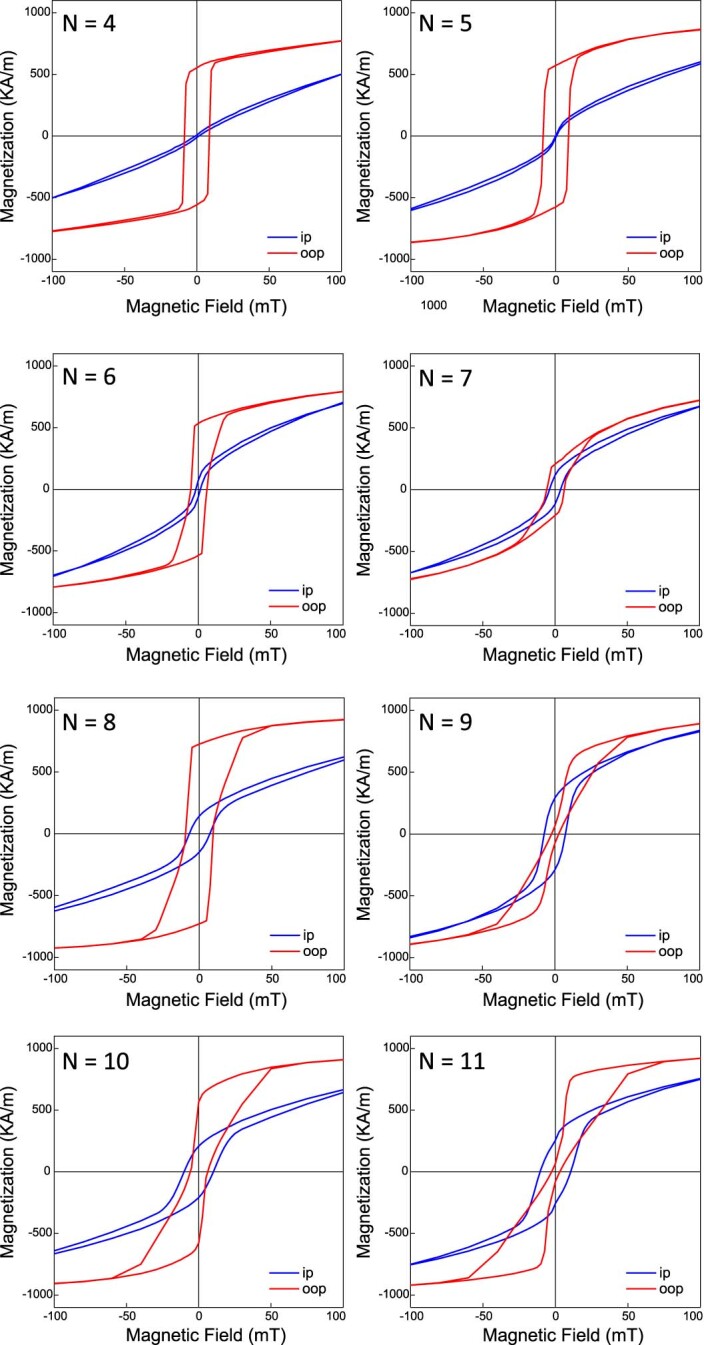
Hysteresis loops. Room temperature *M – H* hysteresis loops of the [Co/Ni]_N_ multilayer sample series where the magnetic field is applied along both OOP and IP directions. Source data

**Extended Data Fig. 4:**
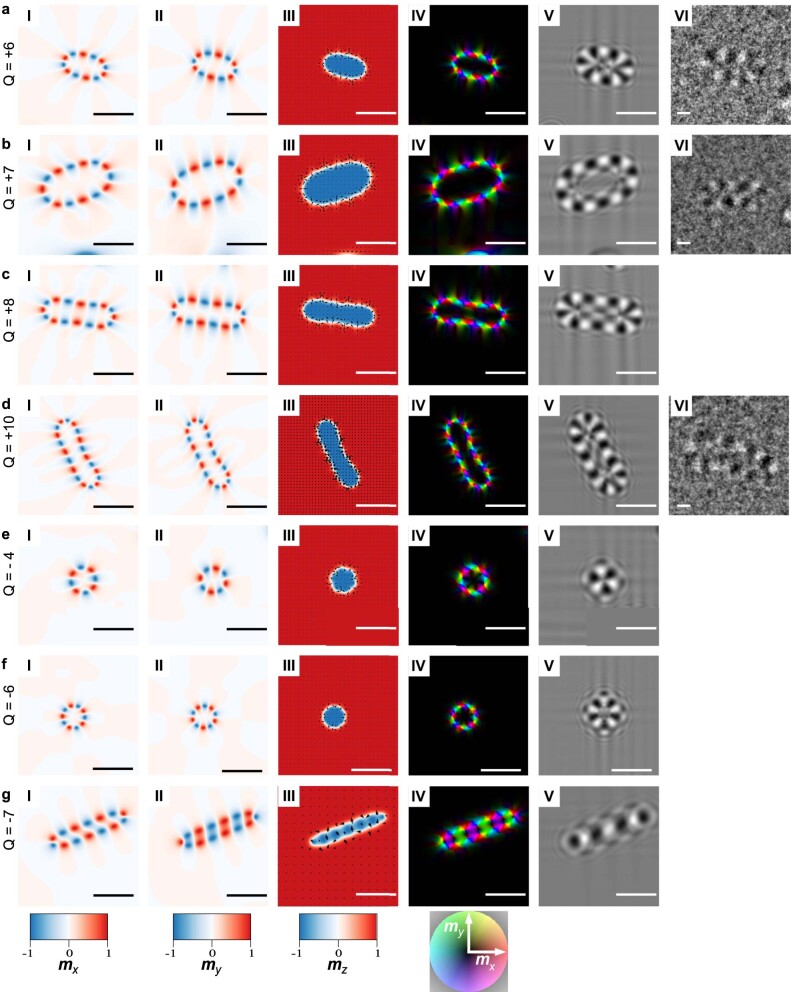
Isolated high-order skyrmions and antiskyrmions. Further high-order ASKs (*Q* = +6 (**a**), *Q* = +7 (**b**), *Q* = +8 (**c**) and *Q* = +10 (**d**)) and SKs (*Q* = −4 (**e**), *Q* = −6 (**f**) and *Q* = −7 (**g**)) have been observed both experimentally and in micromagnetic simulations. **(I)**, *x*, **(II)**, *y*, and **(III)**, *z* components of the magnetization, as well as **(IV)**, magnetic induction maps and **(V)**, simulated LTEM contrast are illustrated for ASKs up to *Q* = 10 and SKs up to *Q* = −8. The corresponding experimental LTEM images are reported in **(VI)**. So far, a *Q* = −9 SK could not be found within our simulations. It is observed that the higher the order, the larger the objects become, being a consequence of the increasing number of Bloch and Néel wall iterations. The scale bars correspond to 100 nm.

A series of [Co(0.2 nm)/Ni(0.7 nm)]_*n*_ multilayers with different bilayer repetition number *n* (*n* = 4–11) exhibiting an easy axis of magnetization in the OOP direction (Extended Data Figs. 2 and 3) was investigated by LTEM at room temperature. As the electrons penetrate at normal incidence through the sample, LTEM is sensitive to only the projected in-plane (IP) magnetic induction, and hence to IP Bloch wall components. Figure 2a shows a typical LTEM image of the [Co/Ni]_10_ sample taken in an applied OOP magnetic field *μ*_0_*H*_*z*_ of 27 mT at 2 mm underfocus distance. Clearly, a variety of coexisting randomly distributed spin objects can be observed, disclosing SKs and ASKs up to |*Q*| = 6. Even SKs and ASKs up to a topological charge number of |*Q*| = 10 could be observed (Extended Data Fig. 4). However, some spin objects cannot be easily classified and are marked with pink circles. The observed SKs and ASKs have sizes in the range of 200–500 nm in diameter, which gradually increases with increasing topological charge. Experimentally acquired LTEM images (Fig. 2b–k(I)) and induction maps (Fig. 2b–k(II)) of isolated high-order spin objects are shown in Fig. 2b–k and are accompanied by simulated Lorentz contrasts (Fig. 2b–k(III)) and induction maps (Fig. 2b–k(IV)) obtained from micromagnetic simulations.

It can be observed that the level of symmetry of LTEM contrast of ASKs exceeds the order by one, for example, a *Q* = 3 ASK shows a fourfold symmetry and has four pairs of black and white segments. Comparing the contrast of a *Q* = 1 ASK and a *Q* = −3 SK with circular symmetry, we see in Fig. 2b(I) and (III) and Fig. 2i(I) and (III), respectively, that they seem rather similar. Corresponding induction maps presented in Fig. 2b(II) and (IV) and Fig. 2i(II) and (IV) allow us to clearly distinguish between them: the former clearly has two pairs of Bloch and Néel walls. In addition, we disclose the direct observation of a *Q* = −5 SK exhibiting an elongated shape (Fig. 2k).

## The origin of high-order spin objects

**Fig. 3:**
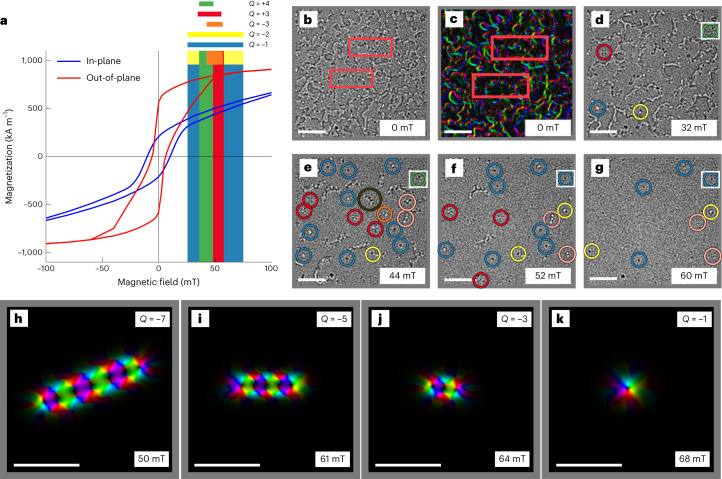
Magnetic domain structure and evolution of various spin objects in an applied OOP magnetic field. **a**, Room temperature OOP and IP *M–H* hysteresis loops of a [Co/Ni]_10_ multilayer including the stability range of different spin objects. **b**,**c**, LTEM image (**b**) at zero field showing domain walls with VBLs highlighted with red boxes, and corresponding induction map (**c**). **d**–**g**, Evolution of the magnetic domain structure at different applied OOP magnetic fields of 32 mT (**d**), 44 mT (**e**), 52 mT (**f**) and 60 mT (**g**). Red and green circles mark *Q* = 3 and *Q* = 4 ASKs, respectively. Blue, yellow, orange and brown circles mark *Q* = −1, −2, −3 and, most likely, *Q* = −9 SKs, respectively. **h**–**k**, Snapshots from micromagnetic simulations illustrated as induction maps showing the field-dependent collapse of a *Q* = −7 SK (**h**), to a *Q* = −5 (**i**), to a *Q* = −3 (**j**) and, finally, *Q* = −1 SK (**k**). Scale bars, 1 µm (**b**–**g**), 200 nm (**h**–**k**). Source data

High-order spin objects do not need to form directly from merging of individual SKs or being encapsulated into SK bags. Let us have a deeper look into their origin. Figure 3a shows the room temperature OOP and IP hysteresis loops of the [Co/Ni]_10_ multilayer. The thin film sample shows the expected easy axis of magnetization along the OOP direction^32–34^ with a saturation magnetization *M*_s_ of about 1,000 kA m^−1^. The OOP loop has a sheared shape with a wide opening typical for Co/Ni multilayers^35^ with perpendicular magnetic anisotropy, which can be attributed to the nucleation/annihilation and growth of OOP polarized magnetic domains parallel to the field. A strong hysteretic reversal behaviour is also observed for the hard axis IP loop. This remanent IP magnetization originates from the polarization of Bloch-type domain walls^32^. Further hysteresis loops of thin film samples for different repetition numbers *n* are given in Extended Data Fig. 3.

Figure 3b,c shows, respectively, the LTEM image and the corresponding induction map of the magnetic domain morphology, disclosing the presence of large OOP magnetic domains separated by Bloch-type domain walls^36–38^ containing VBLs^24^. The magnetic configurations containing VBLs are also schematically depicted in Fig. 1m. These VBLs are described by 180° rotations along the domain wall (red box in Fig. 3b,c), resulting in an inversion of the contrast along a domain wall in the LTEM images^39,40^.

Further LTEM images acquired at different applied OOP fields at the same location while sweeping the field from zero towards magnetic saturation are shown in Fig. 3d–g. When an OOP field is applied, domains with magnetization parallel to the field grow in size, whereas those antiparallel to the field shrink in size. At 32 mT (Fig. 3d), coexisting *Q* = 3 (red circles), *Q* = 4 ASKs (green circles), *Q* = −1 (blue circles) and *Q* = −2 SKs (yellow circles) appear. When the field is further increased to 44 mT, most of the domain walls have vanished, leaving behind various spin objects including *Q* = −3, −4 and −9 SKs (Fig. 3e). At 52 mT (Fig. 3f), most of the spin objects start to vanish and, eventually, at 60 mT only few *Q* = −1 and *Q* = −2 SKs are left (Fig. 3g). By analysing many different field cycles on various samples, one can state that, the higher the charge number or order, the less stable the ASKs, whereas SKs are the most stable ones in our thin film samples. This is in very good agreement with the energy dependence shown in Fig. 1o. Large-scale micromagnetic investigations show that the SKs are indeed stable over the largest field range. However, this can also be attributed to the fact that higher-order SKs are collapsing into energetically most favourable first-order SKs with *Q* = −1 (Fig. 1o), once a critical field is reached. Figure 3h–k illustrates how a *Q* = −7 SK collapses into a *Q* = −1 SK up increasing the OOP field. A similar field-driven collapse of spin objects is also observed experimentally, whereby a *Q* = 4 ASK (Fig. 3d) reduces to a *Q* = −1 SK (Fig. 3f,g) (follow white squares). From simulations, it is apparent that the topological charge decays to the next in odd numbers. If we start with an even number elongated SK, and relax the magnetization, the final state is a circular object for large enough magnetic fields, as shown in the induction maps from Extended Data Fig. 1, and Supplementary Videos 1–16 of an elongated SK with *Q* = −5. In this case the elongated SK widens when the connecting domain walls inside the elongated object collapse into the polarized state forming, finally, a circular SK. Therefore, we expect that, at certain fields, even charged SKs have higher energy; thus, they cannot be achieved by means of field-induced annihilation. This behaviour is fundamentally different from that of composite SKs, whereby one SK after another is annihilated during the field-driven transformation^16,17^. However, a more detailed theoretical study on the energetical stability of the higher-order SKs and ASKs is required to fully understand these topological transformations.

**Extended Data Fig. 5:**
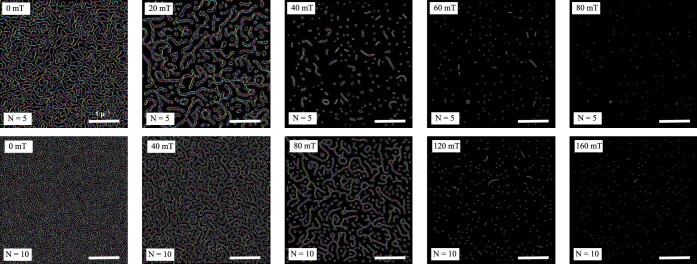
Micromagnetic simulations of large domain structures. Evolution of the magnetization states illustrated by induction maps (colour-wheel as in Fig. 4) obtained by micromagnetic simulations. The snapshots are taken at different values of the externally applied OOP magnetic field. At zero field the domain walls contain a large number of vertical Bloch lines for different film thicknesses corresponding to the [Co/Ni]_N_ multilayer samples with N = 5 and 10. With increasing magnetic field the domains shrink in size, and energetically favoured magnetic quasi-particles form. The scale bars correspond to 1 µm. For details about the simulation methods and used material parameters see Methods section.

**Extended Data Fig. 6:**
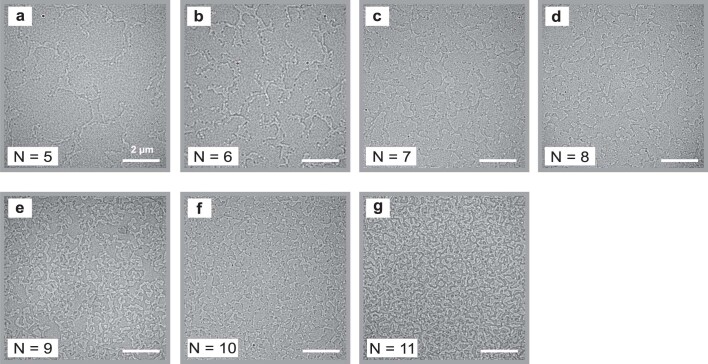
Domain wall configuration. LTEM images of the different [Co/Ni]_N_ multilayers (N = 5 (**a**), N = 6 (**b**), N = 7 (**c**), N = 8 (**d**), N = 9 (**e**), N = 10 (**f**) and N = 11 (**g**)) taken in zero magnetic field. The scale bars correspond to 2 µm.

Full-scale induction maps of simulated structures are given in Extended Data Fig. 5 for two different [Co/Ni]_*n*_ multilayers with *n* = 5 and 10, and sets of material parameters (see Methods for more details). Despite extensive efforts, many observed spin objects could not be classified. For instance, the spin objects highlighted with pink circles in Fig. 3e,f look similar to a *Q* = −5 SK or a *Q* = 4 ASK, but the LTEM contrast does not allow us to confidently classify these spin objects. Note that, for the samples with the lowest repetition numbers (*n* = 4 or 5), only a very weak LTEM contrast could be detected, which did not allow for further investigations (Extended Data Fig. 6).

## Nucleation processes and stability phase diagrams of high-order SKs and ASKs

**Fig. 4:**
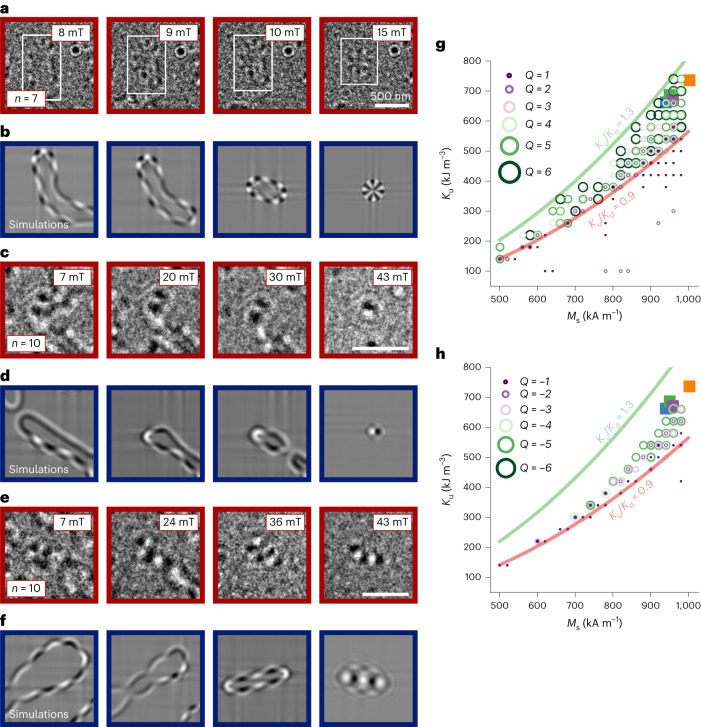
Nucleation process and stability phase diagram of high-order SKs and ASKs. **a**–**f**, Experimentally acquired (**a**,**c**,**e**) and simulated (**b**,**d**,**f**) LTEM images of two [Co/Ni]_*n*_ multilayers (*n* = 7, 10) showing the nucleation process of a *Q* = 6 ASK (**a**,**b**), a *Q* = −2 SK (**c**,**d**) and a *Q* = −5 SK (**e**,**f**). Scale bars, 500 nm. **g**, Stability phase diagram of high-order ASKs depending on the uniaxial magnetic anisotropy *K*_u_ and saturation magnetization *M*_s_. If the coloured circles in the legend are present at a specific (*K*_u_, *M*_s_) coordinate, then the corresponding spin object is stable. In addition, isolines for fixed values of the quality factor ${K}_{\rm{u}}/{K}_{\rm{d}}\left({K}_{\rm{u}}/\left(0.5{\mu }_{0}{M}_{\rm{s}}^{2}\right)\right)$ were included, demonstrating that the optimal quality factor falls within a finite range between 0.9 (red) and 1.3 (green), to ensure the coexistence of high-order SKs and ASKs. The coloured squares correspond to the experimental results. **h**, As in **g** but for high-order SKs. Source data

LTEM imaging and micromagnetic simulations were used to investigate in more detail the nucleation processes of high-order ASKs. A typical nucleation process is given by the *Q* = 6 ASK, which was observed in a [Co/Ni]_7_ sample (Fig. 4a). Micromagnetic simulations and the simulated Lorentz images (Fig. 4b) provide a similar process, whereby we observe that domain walls with a large number of VBLs are shrinking in size with increasing OOP field, leading ultimately to a *Q* = 6 ASK. That is, at 8 mT, a domain wall with four VBLs is present. At 9 mT, the domain wall starts to shrink while the number of VBLs increases. Further increase of the field leads to a reduction of the domain wall area until an isolated *Q* = 6 ASK forms at 15 mT. Note that the VBLs and the higher-order spin objects are found to be stable if we start from a disordered state. If the micromagnetic simulations are performed in such a way that one starts from the saturated state at high external fields, and reduces the applied field, then the system (without defects) will show a low number of, or no, VBLs; thus, spin objects might not be able to be nucleated.

**Extended Data Fig. 7:**
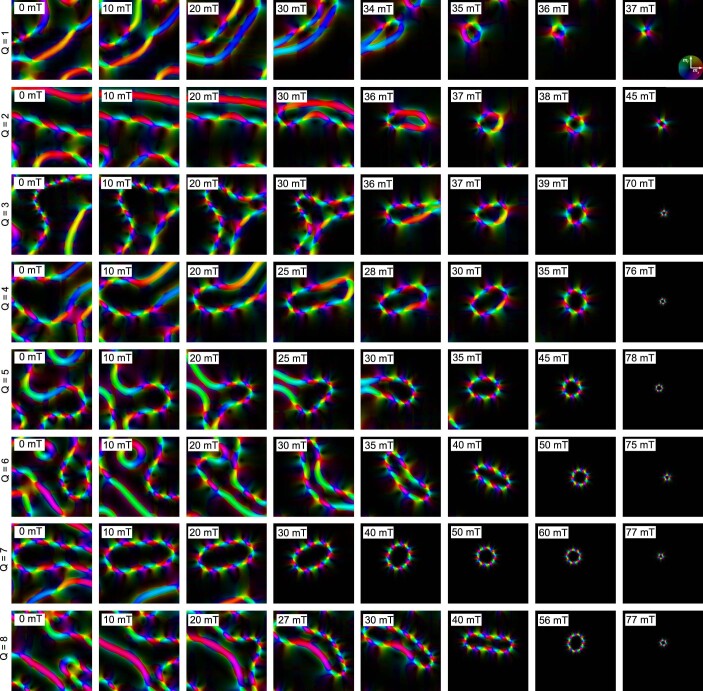
Nucleation of high-order antiskyrmions. Magnetic induction maps illustrating the field dependence of the nucleation process of ASKs up to *Q* = 8. The used film thickness and material parameters correspond to the [Co/Ni]_10_ multilayer sample. The presence of vertical Bloch lines is the essential ingredient in the formation of the high-order ASKs. The color-wheel illustrates the in-plane projection of magnetization.

**Extended Data Fig. 8:**
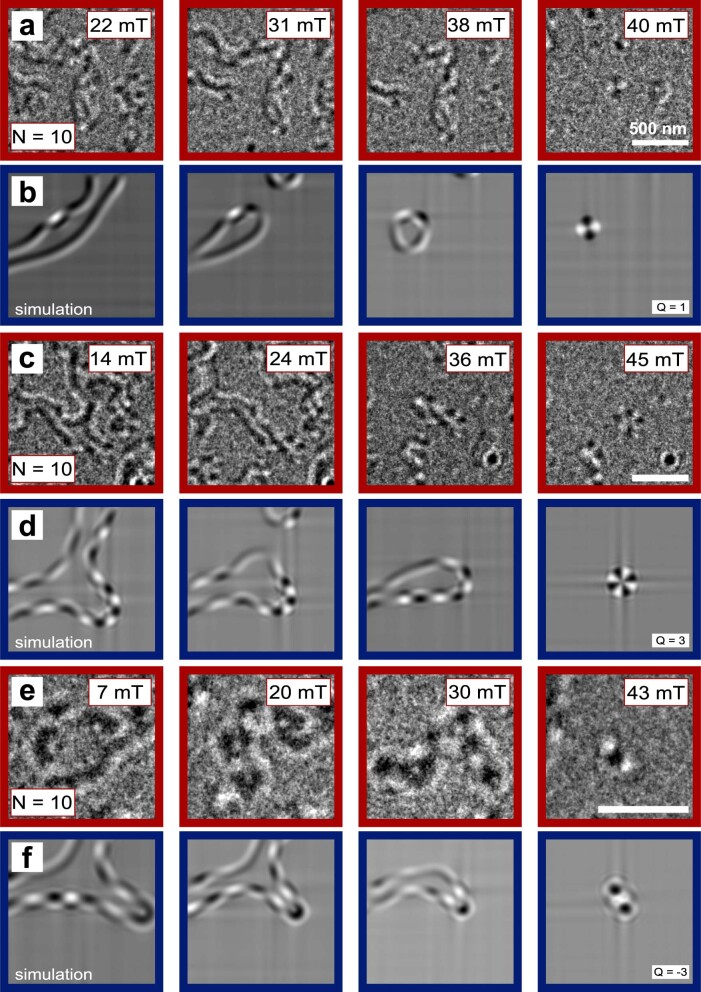
Nucleation process of antiskyrmions and skyrmions. LTEM images (red frames) of the [Co/Ni]_10_ multilayer showing the nucleation process of **a**, *Q* = 1 ASK, **c**, *Q* = 3 ASK, and **e**, *Q* = −3 SK. Below (blue frames) are the corresponding nucleation processes obtained by micromagnetic simulations (**b**, *Q* = 1 ASK, **d**, *Q* = 3 ASK, and **f**, *Q* = −3 SK). The scale bars correspond to 500 nm.

The nucleation processes of high-order SKs were also studied. Figure 4c–f shows the nucleation processes of *Q* = −2 and *Q* = −5 SKs observed in a [Co/Ni]_10_ sample. The LTEM images in Fig. 4c show a domain wall with several VBLs that starts shrinking with increasing field until an isolated *Q* = −2 detaches. The building process is finalized when the SK then detaches from the domain wall, which can be seen as well in the provided Lorentz simulations (Fig. 4d). Similarly, Fig. 4e shows domain walls with a large number of VBLs, and partially formed SKs at 7 mT. At 24 mT, the VBLs start to arrange linearly. Further increase of the field leads to the shrinking and disappearance of the domain wall, leaving behind SKs with opposite chirality. At 43 mT, an isolated *Q* = −5 SK in the form of a linear chain remains, which consists of five SKs with alternating chirality in this elongated spin object. The corresponding simulations show that, initially, a circular object is formed that collapses laterally and joins the ‘half SKs’ into individual SKs and, ultimately, to a *Q* = −5 elongated spin object (Fig. 4f). From these observations it is apparent that high-order ASKs and SKs form from collapsed domain walls with VBLs and that the resulting topological charge depends on the number of involved domain walls and VBLs. However, it is important to note that not all collapsed domain walls with VBLs necessarily nucleate higher-order ASKs and SKs; in some cases, the domain walls just vanish. The nucleation processes for SKs and ASKs of arbitrary topological charge are illustrated in more detail in Extended Data Figs. 1 and 7 (simulations only) and Extended Data Fig. 8 (experiments and simulations).

**Extended Data Fig. 9:**
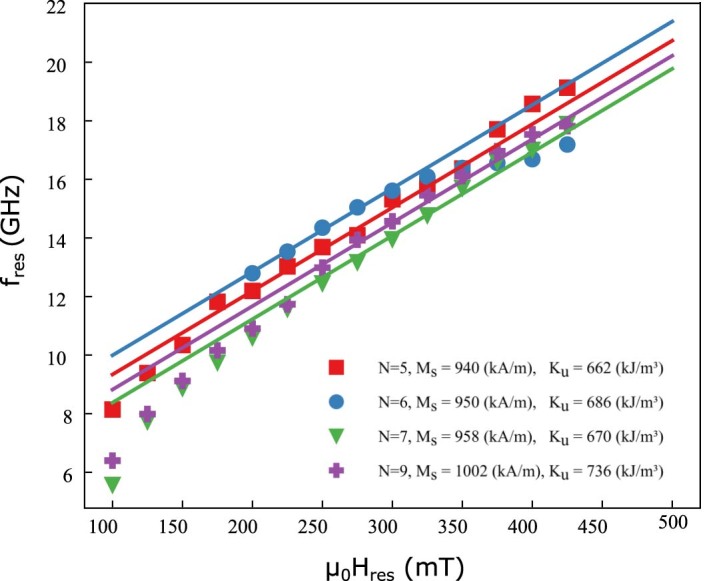
Ferromagnetic Resonance measurements. Field dependency of the resonance frequencies (filled symbols) of the [Co/Ni]_N_ multilayer samples which were fitted using the Kittel formula for the OOP configuration (solid lines), as explained in the Methods section. The fits were done using the *M*_*s*_ values obtained from SQUID-VSM measurements, assuming a constant gyromagnetic ratio, which yield the *K*_*u*_ values given in the legend. Source data

With the aim to better understand the necessary parameter space to stabilize high-order ASKs and SKs, the relevant magnetic properties of the [Co/Ni]_*N*_ sample series were determined. The *M*_s_ values, which are in the range of 1,000 kA m^−1^, were measured by Superconducting Quantum Interference Device – Vibrating Sample Magnetometry (SQUID-VSM) (Extended Data Fig. 3). Ferromagnetic resonance measurements (Extended Data Fig. 9) were performed to extract the uniaxial magnetic anisotropy (*K*_u_) values, which are in the range of 650–750 kJ m^−^^3^, consistent with values reported in the literature^35^. The corresponding experimental *M*_s_ and *K*_u_ values are marked in Fig. 4g,h by coloured squares located at the upper right in the graph.

**Extended Data Fig. 10:**
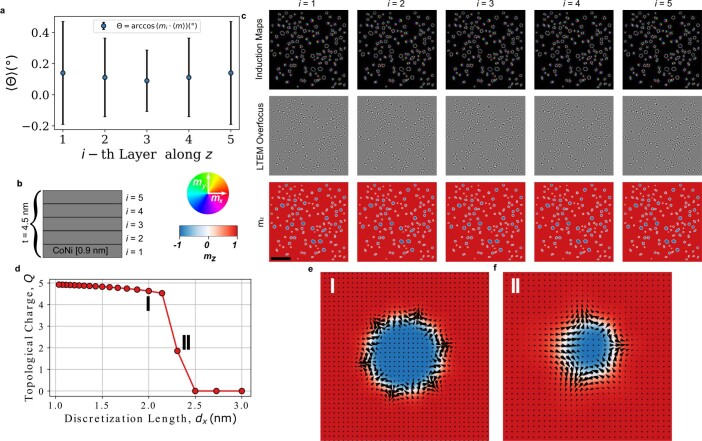
Micromagnetic modeling and validation of continuous model. The multilayer system can be simulated using a continuous quasi 2D model, where the magnetization is assumed to be homogenous along the z direction. Thus, the exchange interaction is assumed to be equal in all three directions. This is a reasonable approximation as the Co and Ni layers are ultra-thin and no spacer layers are involved. The average deviation *θ* from the mean value <*m*> along the OOP direction is smaller than 1 degree (**a**) for the layers marked in (**b**). The total simulation box consists of *n* = *n*_*x* _× *n*_*y* _× *n*_*z*_ = 2000 × 2000 ×5 cells. Thus, 2000 × 2000 vectors were used for each *i*^*th*^ layer to obtain the mean value <*θ*> and the corresponding error bars. If one compares the snapshots of magnetization states, induction maps, as well as LTEM over-focus images for all the layers as plotted in (**c**) then no substantial change can be observed. Scale bars correspond to 500 nm. The more important aspect regarding the correct micromagnetic modeling is the appropriate choice of lateral cell size which is crucial for the correct numerical investigation of the higher order spin textures. In (**d**) the topological charge is plotted against the chosen lateral cell size. For lateral discretization lengths smaller than 2.2 nm a *Q* = +5 ASK can be stabilized, as shown in (**e,I**), when the simulations are started with a rough parametrization of such a higher charged ASK. If the discretization length is increased to 2.25 nm, then the *Q* = +5 ASK decays into a *Q* = +2 type, as shown in (**f,II**). For cell sizes larger than 2.5 nm, no spin objects are stable, and one observes only the polarized ferromagnetic state. Source data

In addition, micromagnetic simulations were performed to investigate the stability range of high-order ASKs and SKs up to |*Q*| = 6 probed at 30 mT depending on the *M*_s_ and *K*_u_ values. The coloured circles in Fig. 4g,h mark the parameter range in which high-order ASKs and SKs are stable. It seems that there exists a clear phase pocket in which a magnetic quality factor ${K}_{\rm{u}}/{K}_{\rm{d}}\left({K}_{\rm{u}}/\left(0.5{\mu }_{0}{M}_{\rm{s}}^{2}\right)\right)$ of about 1 is the crucial parameter to ensure the coexistence of stable solutions with different topological charges, which is in excellent agreement with the experimental results (coloured squares). Note that a very small cell size (<2 nm) is crucial in simulating the high-order ASK and SK, as one needs to resolve the VBLs (Extended Data Fig. 10). The width of this intersegmental domain wall is notably smaller than the regular domain wall width and has to be treated by a very small spatial discretization.

## Current-driven motion of higher-order spin objects

**Fig. 5:**
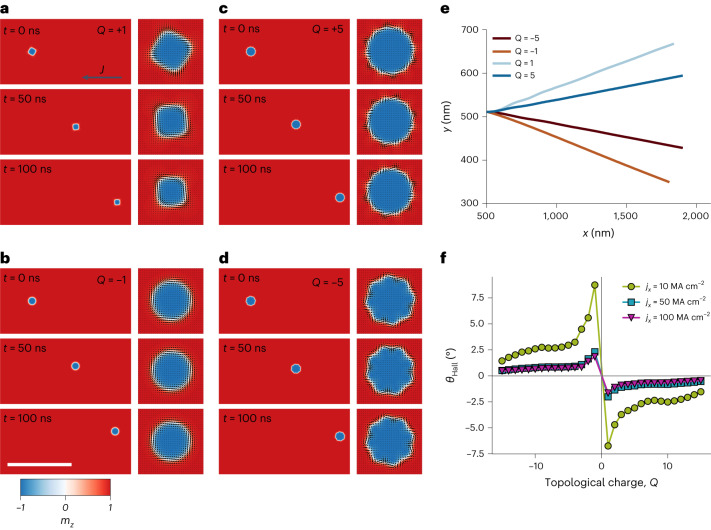
Current-driven motion of high-order SKs and ASKs. **a**–**d**, Snapshots of the magnetization states during the current-driven propagation of (A)SKs with *Q* = +1 (**a**), *Q* = −1 (**b**), *Q* = +5 (**c**) and *Q* = −5 (**d**) obtained from micromagnetic simulations, in which the enlarged objects illustrate the magnetic configuration of the SKs and ASKs. The colour represents the *z* component of the magnetization. The black arrows depict the IP magnetization. Scale bar, 1,000 nm. **e**, The trajectories of the corresponding spin objects until the end of the 100 ns current pulse. **f**, The SK Hall angle *θ*_Hall_ as a function of the topological charge for different applied charge current densities *j*_*x*_, for which the current pulse length was reduced to 25 ns. Source data

A very essential aspect of spin objects is their nature of motion induced by a spin-polarized current^7,28,41^. When a charge current passes through a conducting magnetic material, the polarized electron spin will exert a torque on the magnetization known as the spin-transfer torque. The spin-transfer torque acts only on the local gradient of the magnetization. Thus, in a magnetic system with inhomogeneous magnetization, for example domain walls and SKs, a driving force emerges. Dipolar SKs can then be propagated opposite to the applied charge current. We use micromagnetic simulations to investigate the current-driven motion of *Q* = ±5 and *Q* = ±1 SKs and ASKs; see Methods for more details. Figure 5 illustrates snapshots of the magnetization during the current-driven motion for the four different spin objects. Note that a constant current density of 2 × 10^11^ A m^−2^ was applied under an OOP bias field of 25 mT. The magnetic configurations of the corresponding spin objects are illustrated in the enlarged figures. First, we observe that the spin objects rotate while moving until a steady state is reached, which is very pronounced for the *Q* = 1 ASK. The higher-order SKs and ASKs need less time to enter the steady state and both, SKs and ASKs, remain on a constant trajectory until the end of the 100-ns current pulse (Fig. 5e). Typically, spin objects with integer topological charge different from zero will experience a deviation from the current direction during their spin-transfer torque-induced motion. This effect is known as the SK Hall effect^42^. In our case, this effect is notably reduced with increasing topological charge for SKs and ASKs, as indicated in Fig. 5e. To confirm that the SK Hall effect indeed diminishes for spin objects with higher charge, we performed micromagnetic simulations for spin objects with topologic charge between *Q* = −15 and *Q* = +15 and calculated the SK Hall angle *θ*_Hall_ from the final position of the SKs and ASKs after a current pulse of 25 ns. As shown in Fig. 5f, the highest *θ*_Hall_ angles are observed for *Q* = ±1, which strongly reduce with increasing *Q* value. However, we have to point out that, irrespective of the stabilization mechanism, all magnetic solitons moved by spin-transfer torque will form a circle (or ring) in the velocity plane. The position of a particular SK on that ring depends on many factors, including the ratio of the damping parameter and the coefficient of non-adiabaticity, the topological charge and the symmetry of the SK, as discussed in ref. ^43^. Furthermore, this behaviour depends also strongly on the applied charge current density, which can further help to decrease *θ*_Hall_ (Fig. 5f). Thus, higher-order spin objects provide another route to reduce the SK Hall angle and enable promising advantages over regular SKs, which might be important for future skyrmionic devices.

## Conclusions and discussion

In summary, we have observed for the first time, to our knowledge, high-order SKs and ASKs with *Q* values up to 10 in Co/Ni multilayers. Various spin textures can coexist in this thin film system at room temperature. LTEM images and micromagnetic simulations show that they originate and nucleate from domain walls containing VBLs. A stability phase diagram of high-order SKs and ASKs depending on the uniaxial magnetic anisotropy and saturation magnetization is provided. In this regard, certain highly nonlinear applications such as reservoir computing might profit substantially from the very large number of distinct spin objects existing within the same magnetic system that can interact in a very nonlinear manner, resulting in a non-correlated system for unconventional computing. Furthermore, the numerically demonstrated current-driven motion has shown that the SK Hall angle can be minimized by using higher-order spin textures. This discovery anticipates great potential for further studies and device applications. Thus, we believe that this work opens up an exciting field, whereby the discovery and understanding of spin objects with arbitrary topological charge will help advance several subdisciplines ranging from basic research of topological charge to skyrmionic applications.

## Methods

### Sample preparation and characterization

A series of [Co(0.2 nm)/Ni(0.7 nm)]_*n*_ multilayers with different repetition number *n* (*n* = 4–11) was prepared on both thermally oxidized Si(100) substrates and on 30-nm-thick Si_3_N_4_ membranes required for LTEM imaging. The multilayer films were deposited at room temperature by dc magnetron sputtering. The sputter process was carried out using an Ar working pressure of 3.5 µbar in an ultra-high vacuum chamber. For all samples, a 3-nm-thick Pt seed layer and a 3-nm-thick Si_3_N_4_ capping layer were used to protect the films from oxidation. An illustration of the Pt(3)/[Co(0.2)/Ni(0.7)]_*n*_/Co(0.2)/Ru(0.4)/Si_3_N_4_(3) (all thicknesses are given in nm) multilayer is shown in Extended Data Fig. 2. Note that the prepared Co/Ni multilayer samples exhibit a perpendicular magnetic anisotropy with a quality factor *K*_u_/*K*_d_ ≈ 1, where *K*_d_ is the magnetic shape anisotropy.

### Magnetic characterization

The magnetic properties of the samples were measured by SQUID-VSM. *M*–*H* hysteresis loops were measured in both OOP and IP configurations at room temperature (Extended Data Fig. 3).

### LTEM imaging

The magnetic domain morphology was imaged by LTEM at room temperature using a JEOL NEOARM-200F system operated at 200 keV beam energy in the Fresnel mode with an underfocus of 2 mm. Images are recorded with a Gatan OneView camera. Magnetic induction maps were produced by solving the transport-of-intensity equation using a series of overfocused and underfocused Fresnel images^44^.

### Ferromagnetic resonance measurements

Ferromagnetic resonance measurements were performed using a Quantum Design Physical Property Measurement System, using a probe-holder with a coplanar waveguide. We applied the magnetic field perpendicular to the film plane to investigate the dependence of the resonance frequency (*f*_res_) on the OOP resonance fields (*H*_res_). A Rohde–Schwarz Z40A Vector Network Analyzer was used to investigate the scattering parameter *S*_12_ as a function of frequency and applied magnetic field by sending a microwave signal with 0 dBm power. We performed the measurements with fixed magnetic field and frequency sweep mode at room temperature. The broad linewidth of the resonance peaks made the measurements challenging, but the signal is strong enough to fit with a Lorentzian curve and extract approximately the resonance frequency for different applied magnetic fields after subtracting the background signal. We applied the Kittel formula to extract *f*_res_ as a function of *H*_res_:

$${f}_{\text{res}}=\frac{\gamma {\mu }_{0}}{2\pi }\left({H}_{\text{res}}-{M}_{\rm{s}}+\frac{2{K}_{\rm{u}}}{{{\mu }_{0}M}_{\rm{s}}}\right)$$

where *γ* is the gyromagnetic ratio, *μ*_0_ is the vacuum permeability, *M*_s_ is the saturation magnetization and *K*_u_ is the uniaxial magnetic anisotropy constant. Because we know the saturation magnetization of the samples from the SQUID-VSM measurements, we can extract the anisotropy constant from the Kittel fits. Note that the samples are very thin, thus very weak signals are obtained. But we are interested only in the location of the maxima; thus, this technique is sufficient to obtain reasonable values (Extended Data Fig. 9)

### Micromagnetic simulations

The Co/Ni multilayers were investigated using magnum.np (https://gitlab.com/magnum.np/magnum.np), a PyTorch-based graphics processing unit accelerated finite-difference micromagnetic software that solves numerically the Landau–Lifshitz–Gilbert (LLG) equation taking into consideration different energy contributions^45^. For the investigation of the higher-order spin objects, we consider only contributions of the demagnetization, exchange, uniaxial magnetic anisotropy and external magnetic fields. Note that the appropriate choice of the cell size is crucial here, as shown in the Extended Data Fig. 10

For the numerical investigations, a 5,000 × 5,000 × 1 grid was used with cell sizes of 1 nm × 1 nm × *t* nm, where *t* is the total film thickness of the sample. For the film with *n* = 5 (*t* = 4.5 nm, the following parameters are assumed: *M*_s_ = 940 kA m^−1^, *K*_u_ = 575 kJ m^−^^3^ and exchange stiffness *A*_ex_ of 10 pJ m^−1^. For the thicker sample with *n* = 10 (*t* = 9 nm), *M*_s_ = 1,000 kA m^−1^, *K*_u_ = 675 kJ m^−3^ and A_ex_ = 10 pJ m^−1^ are used. Note that here we are assuming an effective exchange constant that is equal along all three dimensions. In reality, the exchange interaction might be different in different directions. In particular, the IP and OOP directions might have distinct values within an exchange tensor. However, in our ultra-thin layers of Co and Ni, we expect *A*_ex_ to be nearly constant. The lack of spacer layers is crucial for this assumption. Furthermore, we do not use any thermal fluctuations in our numerical modelling. Instead, the above-mentioned magnetic parameters are used as effective temperature-dependent material parameters at *T* = 300 K. All relevant magnetic parameters, *M*_s_, *A*_ex_ and *K*_u_, are temperature dependent. Thus, it is common to perform micromagnetic simulations with effective temperature to qualitatively describe a magnetic system^46,47^. *M*_s_ can be further tuned by the thickness of the individual layers, and *K*_u_ can be tuned by varying the number of Co/Ni repetitions^35^. Thus, we can keep *M*_s_ constant while varying *K*_u_ to study the stability of higher-order spin objects.

The system is initialized with a cell-wise random magnetization, and then the structure is relaxed at vanishing external field. The relaxation process is repeated with different random seeds, to find a good initial state with a high number of VBLs. This process is continued by applying a time-dependent magnetic field along the OOP direction, which increases by 1 mT per 1 ns, until the sample is fully saturated and all the spin objects have vanished. Magnetization states, simulated LTEM images and magnetic induction maps are generated with the built-in functions of magnum.np to visually analyse the spin objects. The obtained magnetization states at each field are investigated and searched for different high-order ASKs. The nucleation processes are then investigated with these simulations (Extended Data Figs. 1, 5 and 7).

To study the stabilization regimes of the high-order ASKs, the above results are used to isolate the magnetic spin textures with orders between 1 and 10. The isolated objects are relaxed in a smaller box with 300 × 300 × 1 cells, with slightly changed applied magnetic fields. Starting from this stable configuration, we add some noise to the system to be sure that we do not end in local minima. The magnetic parameters *M*_s_ and *K*_u_ are varied while keeping *A*_ex_ = 10 pJ m^−1^ constant. A thickness of *t* = 4.5 nm is used. In each simulation, the LLG is solved for 20 ns to be sure that the magnetic textures are not just pinned, but truly stable.

For reproducibility of the presented data of micromagnetic simulations, we performed a double-check with the open-source software Mumax3 (ref. ^48^) and proprietary software Excalibur (http://quantumandclassical.com/excalibur/). In both cases, the stable configurations were obtained by direct energy minimization with different gradient methods. All the data obtained in Mumax3 and Excalibur, including energy values, range of stability and simulated LTEM images (Excalibur only), show excellent quantitative agreement with the data obtained with magnum.np.

The total charge *Q*_tot_ of the entire box is calculated using

$${Q}_{{\rm{tot}}}=\int \frac{1}{4\uppi }m\cdot \left(\frac{\partial m}{\partial x}\times \frac{\partial m}{\partial y}\right){\mathrm{d}}x\,{\mathrm{d}}y$$

By applying a contour-finding algorithm, the total charge *Q*_tot_ is divided by the number of contours, to be sure that the calculated charge is originating from a single spin object.

### Current-driven dynamic simulations

We used a slightly different geometry to simulate the current-driven motion of the higher-order spin objects. A long rectangular geometry of dimensions *l*_*x*_ × *l*_*y*_ × *l*_*z*_ = (4,096 × 1,024 × 5) nm^3^ is discretized using cuboids with volume *d*_*x*_ × *d*_*y*_ × *d*_*z*_ = (2 × 2 × 5) nm^3^. The material parameters used are the same as above for *n* = 5. That is, *M*_s_ = 940 kA m^−1^, *K*_u_ = 575 kJ m^−3^ and *A*_ex_ = 10 pJ m^−1^. A high damping of *α* = 0.1 was used.

A dipolar SK (ASK) is parametrized at *x*_0_ = 500 nm and *y*_0_ = 512 nm. We chose only charges *Q* = ±1 and *Q* = ±5 to demonstrate the current-driven motion. The spin-transfer torque is modelled in magnum.np using the Zhang–Li model, whereby the explicit LLG is completed by an extra torque given by

$$T=-{bm}\times \left[m\times \left(\,{j}_{\rm{e}}\cdot \nabla \right)m\right]-b\zeta m\times \left(\,{j}_{\rm{e}}\cdot \nabla \right)m$$

where *j*_e_ is the applied charge current density. Both *ζ* and *b* are material-specific parameters. The former describes the degree of non-adiabaticity, and the latter is given as

$$b=\frac{\beta {\mu }_{\rm{B}}}{e{M}_{\rm{s}}\left(1+{\zeta }^{\,2}\right)}$$

where *e* is the elementary charge, *μ*_B_ is the Bohr magnetron and *β* is the polarization rate of conducting electrons exerting a torque on the magnetization. For our numerical investigations, we assumed *ζ* = 0.05 and *b* = 72.17 × 10^−12^. The extended LLG was then integrated over 100 ns at a constant current density magnitude of | *j*_e_| = 2 × 10^11^ A m^−2^ while an OOP bias field of *B*_*z*_ = 25 mT was applied.

### LTEM simulation and magnetic induction maps

For the simulation of the magnetic induction maps, and defocused LTEM images, the method of Beleggia and Zhu is used^49^ to calculate the magnetic phase shift given as

$${\tilde{\phi }}_{\rm{m}}({{\mathbf{k}}}_{x},{{\mathbf{k}}}_{y})=\frac{ie{\mu }_{0}{{\mathbf{k}}}_{\perp }}{h}\frac{\tilde{{M}_{\rm{I}}}({{\mathbf{k}}}_{x},{{\mathbf{k}}}_{y})\times {{\mathbf{k}}}_{\perp }}{{{\mathbf{k}}}_{\perp }^{2}}$$

where ${\tilde{\phi }}_{\rm{m}}$ is the integrated magnetization along the thickness, **k** is the **k** vector in Fourier space, *h* is Planck’s constant and *e* is the electron charge. From the magnetic phase shift, one can calculate directly the magnetic induction map

$${B}_{\perp }=\frac{{\varPhi }_{0}}{\pi t}\left(\frac{-\partial {\phi }_{\rm{m}}}{\partial y},\frac{\partial {\phi }_{\rm{m}}}{\partial x}\right)$$

where *Φ*_0_ is the magnetic flux quantum and *t* is the film thickness of the sample.

In addition to the induction maps, the defocused Fresnel images can be generated by means of a convolution theorem, whereby the wavefunction of the electrons is described by ${\psi }_{0}={e}^{i{\phi }_{\rm{m}}}$. The wavefunction at a given defocus value Δ*f* can then be expressed in Fourier space as

$${\tilde{\psi }}_{\Delta f}=\tilde{{\psi }_{0}}{e}^{2i\pi \lambda {{\mathbf{k}}}^{2}\left(\frac{-1}{2}\Delta f+\frac{1}{4}{C}_{\rm{s}}{\lambda }^{2}{{\mathbf{k}}}^{2}\right)}$$

where *C*_s_ is the spherical aberration coefficient of the microscope, *λ* is the relativistic wavelength of the electron and **k** is the wavevector in reciprocal space. The LTEM contrast intensity is then obtained from ${I}_{\Delta f}={\left|{\psi }_{\Delta f}\right|}^{2}$

## Online content

Any methods, additional references, Nature Portfolio reporting summaries, source data, extended data, supplementary information, acknowledgements, peer review information; details of author contributions and competing interests; and statements of data and code availability are available at 10.1038/s41567-023-02358-z.

### Supplementary information

Supplementary Video 1 Nucleation process of a *Q* = 1 antiskyrmion.

Supplementary Video 2 Nucleation process of a *Q* = 2 antiskyrmion.

Supplementary Video 3 Nucleation process of a *Q* = 3 antiskyrmion.

Supplementary Video 4 Nucleation process of a *Q* = 4 antiskyrmion.

Supplementary Video 5 Nucleation process of a *Q* = 5 antiskyrmion.

Supplementary Video 6 Nucleation process of a *Q* = 6 antiskyrmion.

Supplementary Video 7 Nucleation process of a *Q* = 7 antiskyrmion.

Supplementary Video 8 Nucleation process of a *Q* = 8 antiskyrmion.

Supplementary Video 9 Nucleation process of a *Q* = −1 skyrmion.

Supplementary Video 10 Nucleation process of a *Q* = −2 skyrmion.

Supplementary Video 11 Nucleation process of a *Q* = −3 skyrmion.

Supplementary Video 12 Nucleation process of a *Q* = −4 skyrmion.

Supplementary Video 13 Nucleation process of a *Q* = −5 skyrmion.

Supplementary Video 14 Nucleation process of a *Q* = −6 skyrmion.

Supplementary Video 15 Nucleation process of a *Q* = −7 skyrmion.

Supplementary Video 16 Nucleation process of a *Q* = −8 skyrmion.

### Source data

Source Data Fig. 1 Statistical source data.

Source Data Fig. 3a Statistical source data.

Source Data Fig. 4 Statistical source data.

Source Data Fig. 5 Statistical source data.

Source Data Extended Data Fig. 3 Statistical source data.

Source Data Extended Data Fig. 9 Statistical source data.

Source Data Extended Data Fig. 10 Statistical source data.

## Data Availability

The data used in this study are available from the corresponding author upon reasonable request. Source data are provided with this paper.

## Code availability

The codes used in this study are available from the corresponding author upon reasonable request.
